# The gut microbiome is associated with brain structure and function in schizophrenia

**DOI:** 10.1038/s41598-021-89166-8

**Published:** 2021-05-07

**Authors:** Shijia Li, Jie Song, Pengfei Ke, Lingyin Kong, Bingye Lei, Jing Zhou, Yuanyuan Huang, Hehua Li, Guixiang Li, Jun Chen, Xiaobo Li, Zhiming Xiang, Yuping Ning, Fengchun Wu, Kai Wu

**Affiliations:** 1 Department of Biomedical Engineering, School of Material Science and Engineering, South China University of Technology, Guangzhou, 510006 China; 2 The Affiliated Brain Hospital of Guangzhou Medical University, Guangzhou Huiai Hospital, Guangzhou, 510370 China; 3 Guangdong Engineering Technology Research Center for Translational Medicine of Mental Disorders, Guangzhou, 510370 China; 4 Guangdong Engineering Technology Research Center for Diagnosis and Rehabilitation of Dementia, Guangzhou, 510500 China; 5 National Engineering Research Center for Tissue Restoration and Reconstruction, South China University of Technology, Guangzhou, 510006 China; 6 Key Laboratory of Biomedical Engineering of Guangdong Province, South China University of Technology, Guangzhou, 510006 China; 7 National Engineering Research Center for Healthcare Devices, Guangzhou, 510500 China; 8 Department of Biomedical Engineering, New Jersey Institute of Technology, Newark, NJ USA; 9 Department of Radiology, Panyu Central Hospital of Guangzhou, Guangzhou, 511400 China; 10 Department of Nuclear Medicine and Radiology, Institute of Development, Aging and Cancer, Tohoku University, Sendai, 980-8575 Japan

**Keywords:** Clinical microbiology, Neuroscience

## Abstract

The effect of the gut microbiome on the central nervous system and its possible role in mental disorders have received increasing attention. However, knowledge about the relationship between the gut microbiome and brain structure and function is still very limited. Here, we used 16S rRNA sequencing with structural magnetic resonance imaging (sMRI) and resting-state functional (rs-fMRI) to investigate differences in fecal microbiota between 38 patients with schizophrenia (SZ) and 38 demographically matched normal controls (NCs) and explored whether such differences were associated with brain structure and function. At the genus level, we found that the relative abundance of *Ruminococcus* and *Roseburia* was significantly lower, whereas the abundance of *Veillonella* was significantly higher in SZ patients than in NCs. Additionally, the analysis of MRI data revealed that several brain regions showed significantly lower gray matter volume (GMV) and regional homogeneity (ReHo) but significantly higher amplitude of low-frequency fluctuation in SZ patients than in NCs. Moreover, the alpha diversity of the gut microbiota showed a strong linear relationship with the values of both GMV and ReHo. In SZ patients, the ReHo indexes in the right STC (*r* = − 0.35, *p* = 0.031, FDR corrected *p* = 0.039), the left cuneus (*r* = − 0.33, *p* = 0.044, FDR corrected *p* = 0.053) and the right MTC (*r* = − 0.34, *p* = 0.03, FDR corrected *p* = 0.052) were negatively correlated with the abundance of the genus *Roseburia*. Our results suggest that the potential role of the gut microbiome in SZ is related to alterations in brain structure and function. This study provides insights into the underlying neuropathology of SZ.

## Introduction

With the advent of sequencing technology, characterization of schizophrenia (SZ) with probing of the underlying gut microbiome can provide abundant clues for the diagnosis and prognosis of SZ^1^. Several previous studies of the gut microbiome in SZ patients revealed that the species composition within the gut of patients with SZ is different from that of normal control subjects (NCs), with varying bacterial taxa driving community separation in each study, and several of these studies have also focused on the relationship between different gut microbiota and clinical characteristics^2–4^. Converging evidence suggests that the gut microbiota communicates with the central nervous system bidirectionally through the microbiome-gut-brain (MGB) axis^5,6^. A dysregulated MGB axis has been reported in many neuropsychiatric disorders, including SZ^7^, depression^8^, bipolar disorder^9^, autism^10^, Alzheimer’s disease^11^, and Parkinson’s disease^12^.

The gut microbiota can control the expression of a variety of neurotrophic factors, such as brain-derived neurotropic factor (BDNF) and glial cell line-derived neurotrophic factor (GDNF), which can affect neural development and the plasticity of the brain^13^. New findings regarding the MGB axis in SZ patients have recently been reported. Nguyen et al.^14^ suggested that SZ is associated with gastrointestinal inflammation. Additionally, gut and digestive disturbances are highly prevalent comorbidities in SZ patients^15^. However, few studies have reported on the relationship between the gut microbiome and brain structure and function.

Magnetic resonance imaging (MRI) techniques have been widely adopted to study abnormalities in brain structure and function in SZ^16^. A meta-analysis found that SZ showed widespread attenuation of the cortical thickness and surface area, especially in the frontal and temporal regions, compared with NCs^17^. Voxel-based morphometry (VBM) analysis found that SZ patients showed reduced gray matter volume (GMV) in insular subregions^18^.

Alterations in brain function were also observed in SZ^19^. As one of the methods used to measure local resting functional connectivity (FC) or synchronization^20^, regional homogeneity (ReHo) represents the most efficient, reliable, and widely used index beyond the different FC metrics^21,22^. The calculation of ReHo assumes that a given voxel is temporally similar to that of its neighbors^23^. Specifically, taking the voxel or vertex of high-resolution connectomes in the graph as a node, the ReHo index of this node is computed as Kendall’s coefficient of concordance (KCC)^23^. Previous studies found that SZ often showed increased ReHo in the inferior and middle temporal areas^22^, the bilateral superior medial prefrontal cortex (mPFC)^20^, the right superior frontal gyrus, the right superior temporal cortex (STC)^24^ and the fusiform gyrus^22^ but decreased ReHo in the right precentral lobule, the right inferior parietal lobule (IPL), the left paracentral lobule^20^, the left postcentral gyrus and the left STG^24^ compared with NCs.

Additionally, the amplitude of low-frequency fluctuation (ALFF) has been widely used as a neuroimaging biomarker to explore resting-state regional brain activity in psychiatric disorders, including schizophrenia^25^. A meta-analysis of resting-state functional MRI (rs-fMRI) reported decreased ALFF in the bilateral postcentral gyrus, bilateral precuneus, left inferior parietal gyri and right occipital lobe, and increased ALFF in the right putamen, right inferior frontal gyrus, left inferior temporal gyrus and right anterior cingulate cortex in SZ^26^.

In the present study, we hypothesized that in SZ patients, the between-group differences in gut microbial composition might be associated with the between-group differences in the GMV, ReHo and ALFF indexes. To this end, we recruited 76 participants, including 38 SZ patients and 38 NCs, and collected their rs-fMRI data and stool samples. Next, 16S rRNA sequencing was applied to analyze the composition of the gut microbiome, the GMV index was calculated to explore brain structure alterations, and the ReHo and ALFF indexes were calculated to explore functional brain activity. Finally, we analyzed the correlation between the gut microbiome and the GMV, ReHo and ALFF indexes.

## Results

### Clinical data

The cohort investigated in this study comprised 38 SZ patients and 38 NCs who did not differ in terms of age, sex, BMI or several other parameters (*p* > 0.05). SZ patients had higher rates of smoking (*p* = 0.03), longer sleep times (*p* = 0.007) and fewer years of education (*p* = 0.01). However, in terms of alcohol intake, there was a higher rate of alcohol consumption in the NC group (*p* < 0.001) (Table 1).

**Table 1:** Demographic characteristics of the NC and SZ patients.

Characteristic	NC (n = 38)	SZ (n = 38)	*p* value
Age (years)	35.47 ± 11.54	35.26 ± 10.76	0.94
Gender (M/F)	22/16	20/18	0.82
BMI (kg/m^2^)	22.63 ± 2.63	23.70 ± 4.54	0.28
Education (years)	14.42 ± 2.93	12.26 ± 4.11	0.01
Sleep time (hours)	7.31 ± 0.73	8.43 ± 1.98	0.007
Alcohol	47.37%	0%	< 0.001
Smoker	2.63%	21.05%	0.03
Diastolic pressure (mmHg)	77.84 ± 8.18	77.26 ± 9.61	0.82
Systolic pressure (mmHg)	118.45 ± 9.93	115.91 ± 17.08	0.54
PANSS positive score	–	10.81 ± 5.50	–
PANSS negative score	–	17.89 ± 8.28	–
PANSS general score	–	27.71 ± 8.23	–
PANSS total score	–	56.95 ± 19.51	–
HDLC (mmol/L)	1.60 ± 0.30	1.54 ± 0.35	0.39
LDLC (mmol/L)	3.37 ± 0.85	3.20 ± 0.83	0.38
Glu (mmol/L)	5.80 ± 1.42	5.23 ± 1.25	0.07

BMI, body mass index; HDLC, high-density lipoprotein cholesterol; LDLC, low-density lipoprotein cholesterol; Glu, glucose.

### Sequencing data and bacterial taxonomic composition

**Figure 1:**
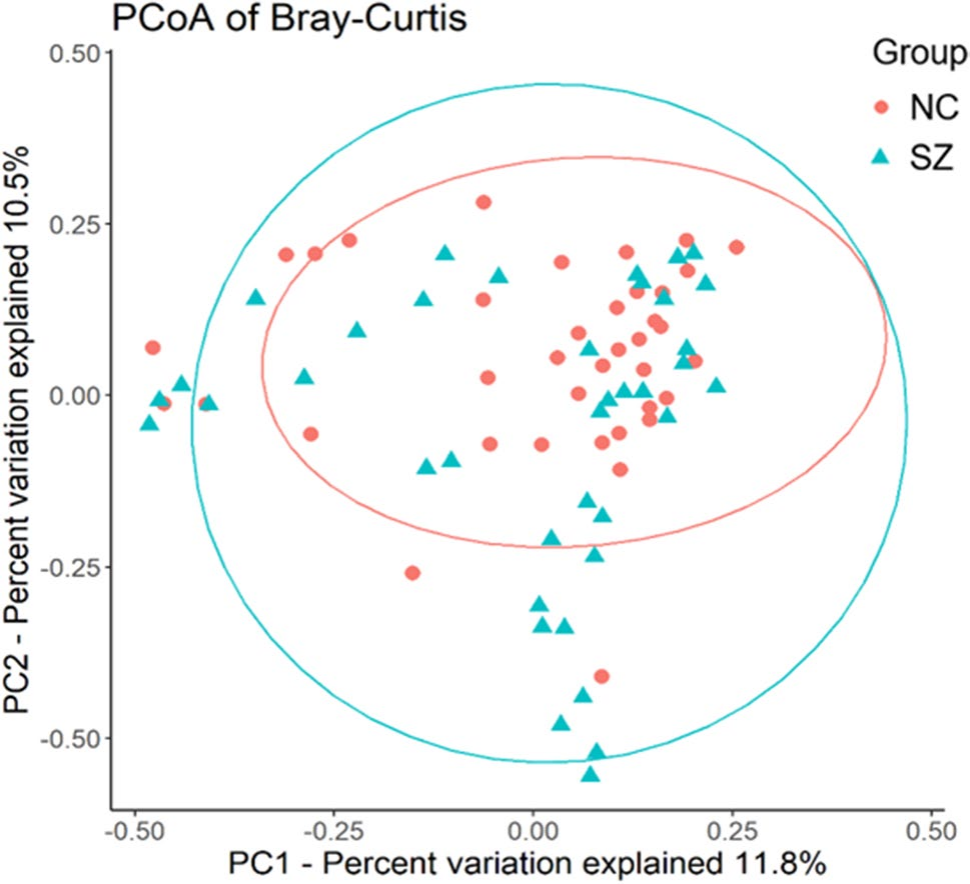
PCoA plot illustrating beta diversity distance matrices of the Bray–Curtis distance comparing the sample distribution between the two groups. The red dots represent NCs, and the green triangles represent SZ.

There was no significant difference between the two groups on any assessed measure of alpha diversity (Supplementary Table S1). To determine whether the overall gut microbiome composition differed between the two groups, we performed principal coordinate analysis (PCoA) of the Bray–Curtis distance. As shown in Fig. 1, we found a significant between-group difference in Bray–Curtis distance (pseudo-F = 1.71, *p* = 0.019) under 999 permutations, and the PCoA of the Bray–Curtis distance showed that the SZ and NC groups formed distinct clusters.

**Figure 2:**
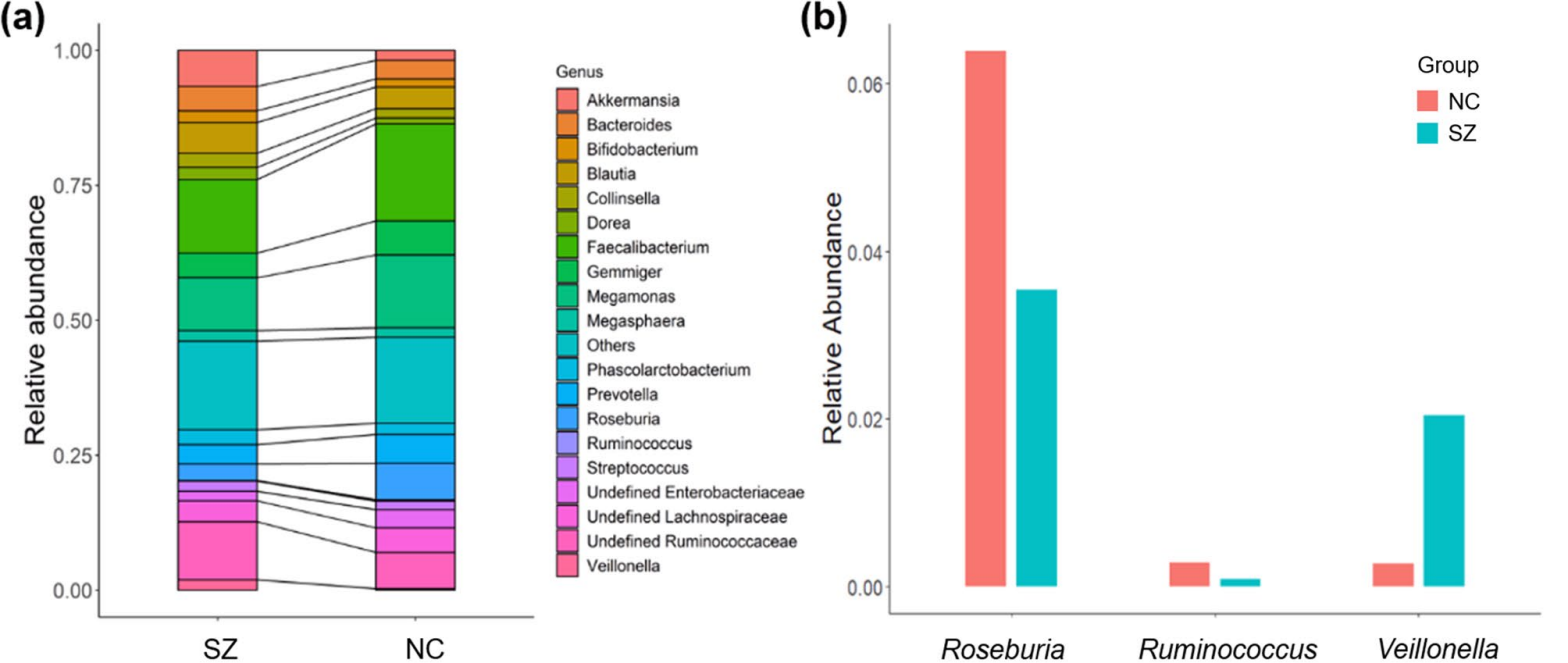
Microbial composition at the genus level. (**a**) Summary of the most abundant genera in the NC and SZ groups. (**b**) Bacterial genera that were significantly different between the two groups (*p* < 0.05, uncorrected).

The bacterial composition results showed that sequences from the NC group were mainly assigned to *Faecalibacterium*, *Megamonas*, *Roseburia* and *Gemmiger* at the genus level; the most abundant genus in the SZ group was also *Faecalibacterium*, followed by *Megamonas*, *Ruminococcus* and *Akkermansia* (Fig. 2a). Compared to those in the NC group, the relative abundances of *Ruminococcus* (*p* = 0.017, uncorrected) and *Roseburia* (*p* = 0.023, uncorrected) were significantly lower in the SZ group, while the relative abundances of *Veillonella* were significantly higher in the SZ group (Fig. 2b).

### Differences in MRI indexes between the SZ patients and NCs

We found significant differences in GMV in 16 brain regions between SZ patients and NCs. Additionally, significant differences in ReHo in 34 brain regions were found between SZ patients and NCs. Only one brain region showed significant between-group differences in ALFF. See the detailed information in Supplementary Table S2 in the Supplementary Materials.

### Relationship with the MRI indexes

**Figure 3:**
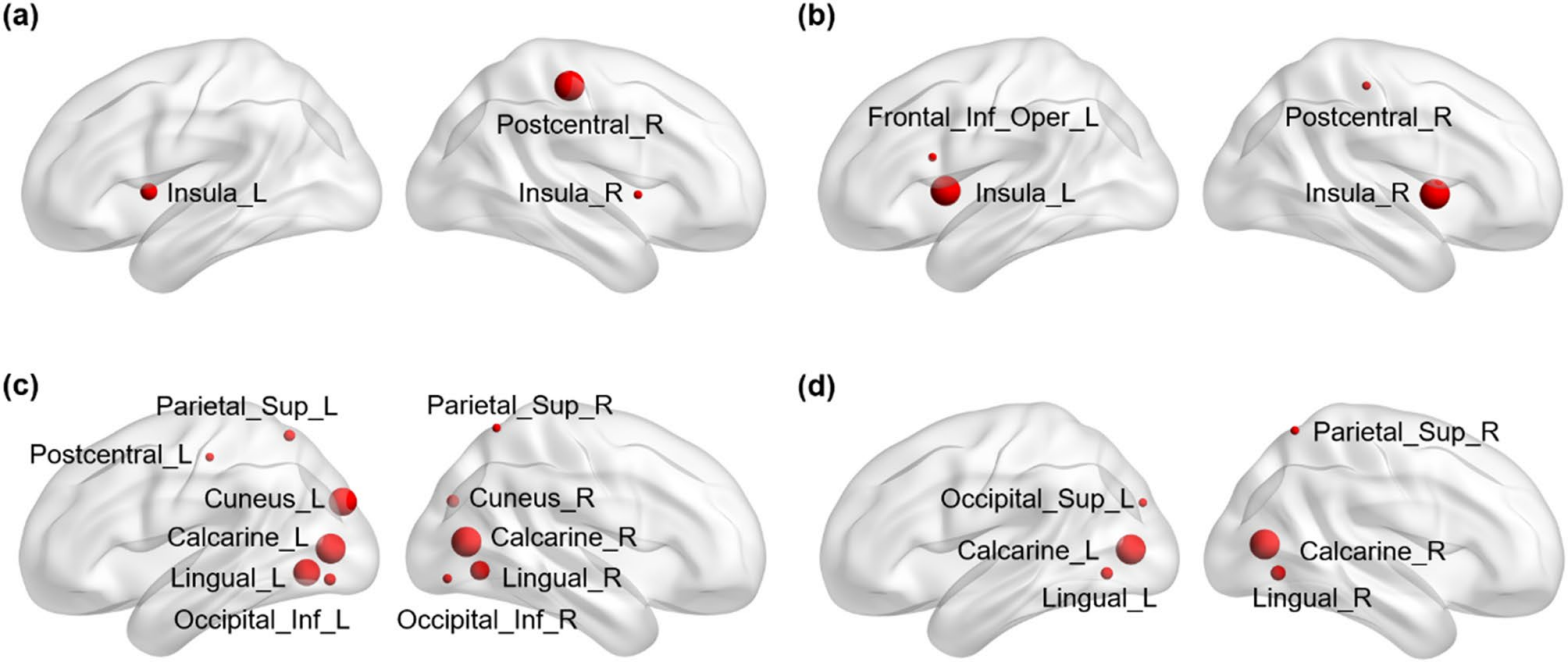
Alpha diversity of gut microbiota in the SZ patients showed strong positive correlations with GMV and ReHo. (**a**) Brain regions showing significant correlations between the residuals of the GMV index and the observed species alpha diversity. (**b**) Brain regions showing significant correlations between the residuals of the GMV index and the Faith_PD of alpha diversity. (**c**) Brain regions showing significant correlations between the residuals of the ReHo index and the evenness of alpha diversity. (**d**) Brain regions showing significant correlations between the residuals of the ReHo index and the Shannon alpha diversity. The size of the node indicates the relative size of the *r* value of the significant correlation; the red color of the node indicates a positive correlation between the residuals of the GMV as well as the ReHo indexes and the residuals of the alpha diversity. Sup: superior; Inf: inferior; L: left hemisphere; R: right hemisphere. Figure was generated by a brain network visualization tools of “BrainNet Viewer” (Version 1.7, https://www.nitrc.org/projects/bnv/), based on MATLAB (Version 2017a).

Figure 3 shows the significant relationship between the diversity of the microbiome and the MRI indexes in the SZ patients. Both the alpha diversity Faith_PD and the observed species were correlated with the GMV of the bilateral insula and right postcentral gyrus (*p* < 0.05). Meanwhile, Faith_PD showed a positive correlation with the GMV of the left inferior operculum frontal cortex (*p* < 0.05). Both evenness and Shannon indexes in the SZ group were positively associated with the ReHo indexes of the bilateral calcarine cortex, bilateral lingual gyrus, left superior occipital cortex and right superior parietal cortex (*p* < 0.05). Additionally, the evenness of alpha diversity showed positive correlations with the ReHo indexes of the right cuneus lobe, bilateral fusiform gyrus, left postcentral gyrus and left superior parietal cortex (*p* < 0.05). No significant correlation was detected between the microbial diversity and the ALFF index. Detailed information on the correlations between the microbial diversity and MRI indexes is listed in Supplementary Table S3.

**Figure 4:**
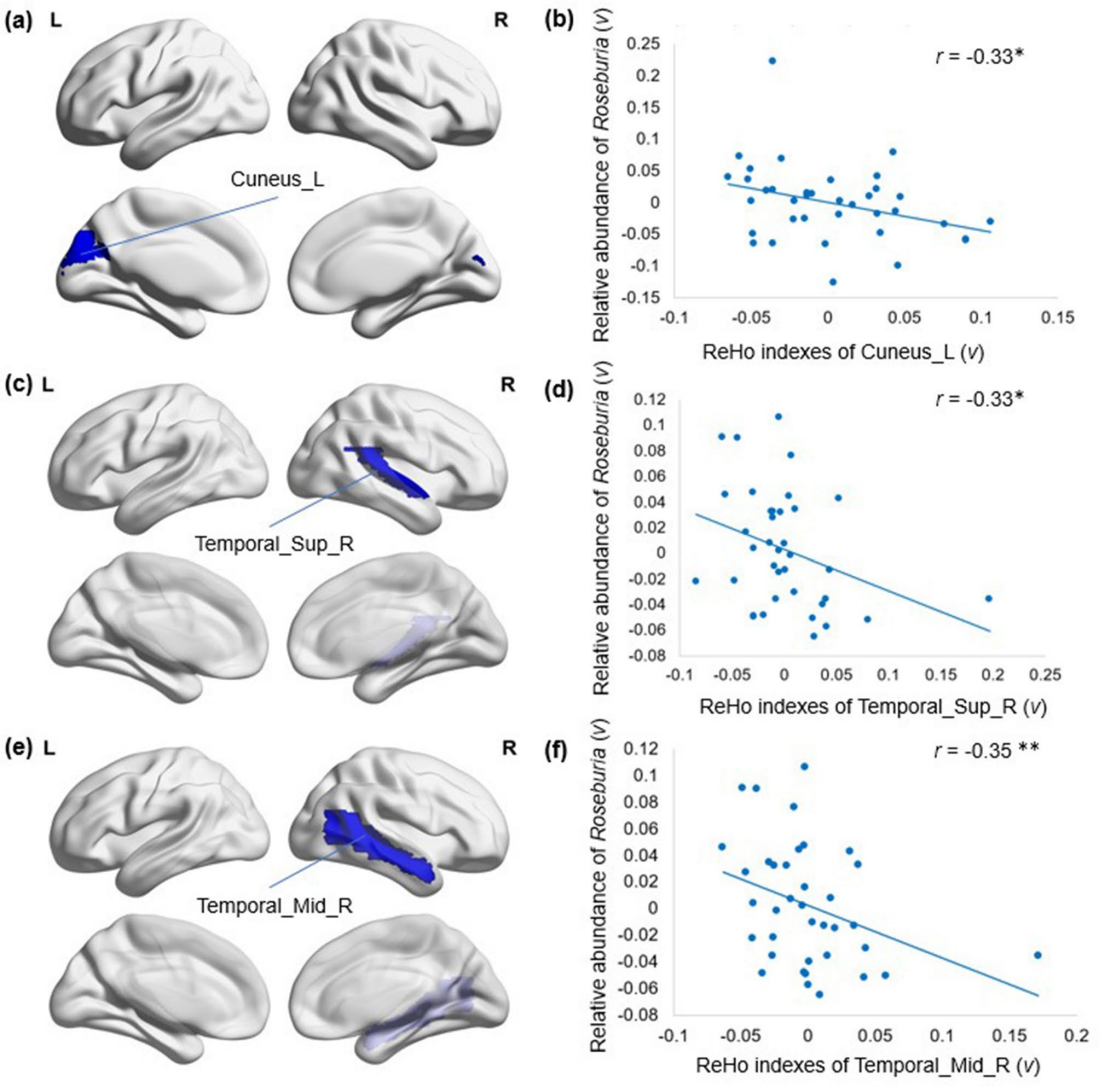
Relative abundance of *Roseburia* in SZ patients showed strong negative correlations with ReHo indexes. (**a**) Cuneus_L showed significantly decreased ReHo indexes in SZ compared with the NCs (*p* < 0.05). (**b**) The residuals of the ReHo indexes in Cuneus_L were significantly negatively correlated with the residuals of the relative abundance of Roseburia in SZ. (**c**) Temporal_Sup_R showed significantly decreased ReHo indexes in SZ compared with the NCs (*p* < 0.05). (**d**) The residuals of the ReHo indexes in Temporal_Sup_R were significantly negatively correlated with the residuals of the relative abundance of *Roseburia* in SZ. (**e**) Temporal_Mid_R showed significantly decreased ReHo indexes in the SZ compared with the NCs (*p* < 0.05). (**f**) The residuals of ReHo indexes in Temporal_Mid_R were significantly negatively correlated with the residuals of the relative abundance of *Roseburia* in SZ. Sup: superior; Inf: inferior; L: left hemisphere; R: right hemisphere. **p < 0.05, FDR corrected, *p = 0.05, FDR corrected. Figure was generated by a brain network visualization tool of “BrainNet viewer” (Version 1.7, https://www.nitrc.org/projects/bnv/), based on MATLAB (Version 2017a).

After identifying both genera and MRI indexes showing significant between-group differences, we further tested for associations between the abundance of each genus and the MRI indexes. In the SZ group, we found that the ReHo indexes in the right STC (*r* = − 0.35, *p* = 0.031, FDR = 0.039), left cuneus (*r* = − 0.33, *p* = 0.044, FDR = 0.053) and right MTC (*r* = − 0.33, *p* = 0.03, FDR = 0.052) were negatively correlated with the abundance of the genus *Roseburia*. The relationships between the relative abundance of *Roseburia* and the ReHo indexes of 3 brain regions are shown in Fig. 4.

## Discussion

To the best of our knowledge, this is the first study to find a correlation between the gut microbiome and the brain structure and function in SZ. The main findings are as follows: (1) Consistent with previous studies, significant between-group differences in the gut microbiota and MRI indexes were found; (2) in SZ patients, both the alpha diversity evenness and Shannon indexes showed a positive correlation with the GMV and ReHo indexes in several brain regions; and (3) in SZ patients, the ReHo indexes in the right STC, left cuneus and right MTC were negatively correlated with the relative abundance of the genus *Roseburia*.

The results from 16S rRNA sequencing demonstrated significant changes in microbial composition between the two groups. The gut microbiome is a complex system that is affected by factors such as diet, age, alcohol consumption and smoking. There is evidence suggesting that exposure to alcohol results in specific modifications of the microbiome composition, bacterial overgrowth and disruption of mucosal barrier function^27^. Chronic alcohol consumption leads to an increase in *Proteobacteria* and a decrease in *Bacteroidetes* and increased intestinal permeability, resulting in translocation of LPS and endotoxins in the bloodstream, which eventually contributes to hepatic damage^28,29^.

Cigarettes produce a large number of compounds that have some kind of deleterious effects on different organs, and some of them exert an effect on intestinal health and on the microbiome. Rogers et al. reported an increased rate of *C. difficile* infection in smokers, with odds 33% greater in former smokers and 80% greater in current smokers than in never smokers^30^. Smoking withdrawal in humans results in profound changes in the microbiome, with increased microbial diversity, an increase in *Firmicutes* and *Actinobacteria* and a decrease in *Bacteroidetes* and *Proteobacteria*^31^.

In this study, shifts in taxonomic abundance in SZ were consistent with previous studies. For instance, *Roseburia* (order *Clostridiales*) and *Ruminococcus* (order *Lactobacillales*) showed depletion in SZ, which was observed in multiple previous studies on psychiatric diseases^4,32,33^. *Roseburia* and *Ruminococcus* are representative bacteria that produce short-chain fatty acids (SCFAs), including butyrate and propionate, which are considered to benefit health^34^. Butyrate is produced from carbohydrates via glycolysis from the combination of two molecules of acetyl‐CoA to form acetoacetyl‐CoA, followed by stepwise reduction to butyryl‐CoA. *Roseburia* can produce butyrate and propionate via the butyrate kinase route and the 1,2-propanediol pathway^35^. However, *Ruminococcus* lacks the ability to form butyrate from carbohydrates. The gut microbiota can produce propionate via sugar fermentation, and the main pathway is the succinate pathway. The succinate pathway is known to be present in *Ruminococcus*, which produces succinate rather than propionate as the end product. On the other hand, some human colonic bacteria belonging to the *Negativicutes* class of *Firmicutes* have the ability to convert succinate to propionate^36^. In addition, *Veillonella* can convert lactate to propionate via the succinate pathway^37^.

Microbial-derived SCFAs can cross the blood–brain barrier (BBB) and activate specific receptors in relevant brain regions pertinent to depression and anxiety-related behaviors^38^. In fact, a decrease in the relative abundance of *Roseburia* may be detrimental to insulin sensitivity and thus affect the concentrations of branched-chain amino acids (BCAAs)^39^. Due to the brain transporters shared between BCAAs and tryptophan, the presence of excessive BCAAs would cause decreased efficiency of the transportation of tryptophan. It has been reported that excessive consumption of BCAAs could lead to decreased cerebral concentrations of tryptophan, the precursor of serotonin, and thus result in decreased cerebral 5-hydroxytryptamine concentrations^40^. Li et al.^41^ speculated that depletion of the *Clostridiales* taxa, which degrade BCAAs, leads to an elevated concentration of BCAAs in the circulatory system and therefore indirectly decreases cerebral serotonin concentrations, which affects mood.

In this study, we found structural abnormalities in SZ. Our results showed that the GMV of some brain regions, including the bilateral insula, frontal and temporal regions (see Supplementary Table S2 for the detailed regions) in SZ patients was decreased compared with that in NCs. These results are in line with previous studies that found that SZ showed decreased GMV in the insula^42^, superior temporal pole^43^, amygdala, anterior cingulate, and frontal cortices (superior, middle, opercular inferior, and orbital frontal gyrus)^42,43^. Van Rheenen et al. reported entire cortex volume reductions in SZ with cognitive impairments^44^. Actually, a larger cortical volume or greater gray matter density in most brain regions is often associated with better computational efficacy^45^. Thus, we inferred that cognitive impairments may be related to the reduction in GMV in SZ.

We found that most brain regions showed lower functional brain activity in SZ patients than in NCs (Supplementary Table S2). This result is consistent with previous studies that reported altered ReHo in SZ in the bilateral STC^46^, the MTC^22,46^, the bilateral superior medial prefrontal cortex (mPFC)^20^, the right superior frontal gyrus (SFG), and the fusiform gyrus^20,22^ compared with those in NCs. Most of the brain regions that showed altered ReHo, such as the STG, MTC and SFG, are related to visual and auditory perception and they are mainly located in the frontal and temporal areas (Supplementary Table S2). Several studies have reported that temporal lobe abnormalities may be related to the emergence of auditory hallucinations^47^, abnormal language processing^47^, thought disorders^48^, and other psychotic symptoms in SZ. Additionally, the frontal lobe mediates a number of important processes that may impact executive function, working memory, abstract reasoning, social behavior, empathy, self-monitoring, and impulse control in SZ^47,49^. We also found that SZ patients showed increased ALFF in the right caudate compared with NCs (Supplementary Table S2). Previous studies reported that SZ patients showed significantly increased ALFF in the right caudate nucleus^50,51^, middle temporal gyrus, inferior parietal lobule^50^, bilateral prefrontal and parietal cortex, and left superior temporal cortex^51^ compared with NCs. These findings suggested that the local synchronization of spontaneous activity and the amplitude of fluctuations in SZ brains was widely disrupted, which could help explain the psychopathology of SZ.

We observed significant positive associations between the gut microbial diversity measures and the reduction in GMV in SZ (Fig. 3). Previous studies found that variations in genes were associated with GMV reduction in SZ, especially in the prefrontal cortex and anterior cingulate cortex^52–54^. Transcription-neuroimaging association analysis found that the expression levels of 98 genes were significantly correlated with GMV changes in SZ^55^. Although no prior study reported the relationship between GMV and the gut microbiome in SZ, decreased structural integrity of both white and gray matter regions, including the hippocampus, was observed in mice that were colonized with attention-deficit/hyperactivity disorder (ADHD) microbiota^56^. One study reported that the relative abundance of *Bacteroides* showed greater prominence in the cerebellum, frontal regions, and hippocampus in women, which further supports the hypothesis that microbial modulation may affect mood and behavior^57^. Interestingly, we also found several brain regions associated with PANSS scores in SZ patients (Supplementary Table S4).

Some species have been reported to be associated with brain function. *Prevotella* has been shown to be associated with the development of brain abscesses and other neurological syndromes via the production of IgA proteases that promote virulence and initiate an immune response^58,59^. Lin et al. reported that the genus *Neisseria* is negatively associated with functional network connectivity (FNC) loading, especially the FNC between the left angular gyrus and right inferior occipital gyrus, which is related to visual processing function^60^. In addition, members of the genus *Neisseria*, including the species *Neisseria meningitidis*, stimulate the immune system through a variety of mechanisms and invade the neurological nervous system during infection^61^. In this study, we found that the depletion of the genus *Roseburia* was significantly associated with the local synchronization of spontaneous activity of the right STC and the right MTC, which are related to auditory verbal hallucinations^62,63^ and thought disturbances^64^ in SZ. Dhiman et al.^65^ reported that *Roseburia* was associated with good cognitive performance, which may further support our findings. A preliminary study on the gut microbiome and brain functional connectivity in infants revealed that alpha diversity was significantly associated with functional connectivity between the amygdala and thalamus and between the anterior cingulate cortex and anterior insula^66^, suggesting a potential pathway linking gut microbial diversity and cognitive outcomes.

We found that both evenness and Shannon of microbial diversity in SZ were positively associated with ReHo indexes of the bilateral calcarine cortex, bilateral lingual gyrus, left superior occipital cortex and right superior parietal cortex. Additionally, evenness showed positive correlations with ReHo indexes of the right cuneus lobe, bilateral fusiform gyrus, left postcentral gyrus and left superior parietal cortex (Table S3). We inferred that the sensory and cognitive impairments might be related to the alteration of ReHo indexes and microbial alpha diversity in SZ. For example, gut microbial alpha diversity in SZ may be associated with visual hallucinations^62,67^, and this relationship could be mediated by functional brain activity in the calcarine cortex.

The present study has several limitations. First, the SZ varied considerably in their medications. Specifically, the durations and types of medication for SZ were distinct. It is important to note that medication can affect microbiota composition and brain activity, which may further affect the results. Second, although we detected correlations between the MRI indexes and microbiota composition as well as diversity in the SZ group, we cannot determine the causal relationship between them. A future longitudinal study may contribute to solving this problem. Third, because the rates of smoking and alcohol consumption were low among all participants and detailed information was not collected, this study could not determine the effects of these two factors on the gut microbiome. Finally, the sample size was moderate. A larger independent sample is needed to examine the reproducibility of our findings.

## Methods

### Participants

A total of 76 subjects were recruited, including 38 SZ patients recruited from the Affiliated Brain Hospital of Guangzhou Medical University and 38 age-, sex- and BMI-matched NCs recruited in Guangzhou and surrounding areas. The diagnosis of SZ was made based on the Diagnostic and Statistical Manual of Mental Disorder-IV-Text Revision (DSM-IV-TR) (SCID). Subjects with stable psychiatric symptoms for > 2 weeks and a total Positive and Negative Syndrome Scale (PANSS) score of ≥ 30 with a rate of change of ≤ 20% at 2 weeks were included in the study. Thirty-five of the SZ patients were on antipsychotic medication at the time of the study. The exclusion criteria for all participants included (1) any other current major DSM-IV-TR Axis I diagnoses; (2) any somatic diseases; (3) a history of epilepsy, except for febrile convulsions; (4) a history of having received electroconvulsive therapy in the past 6 months; (5) lactating, pregnant, or planning to become pregnant; (6) alcohol dependence or (7) noncompliance with drug administration or a lack of legal guardians.

The study protocol was approved by the ethics committees of the Affiliated Brain Hospital of Guangzhou Medical University, and written informed consent was obtained from each subject or their legal guardian prior to the study. A questionnaire was conducted among all subjects to collect general information, including age, sex, BMI, years of education, history of medication used and history of smoking and drinking.

### Fecal sample collection and processing

Fresh fecal samples were collected from participants after fasting for 12 h, and all of the samples were stored at − 80 °C until DNA extraction. A total of 200 mg of each fecal sample was used for DNA extraction. The method of DNA extraction was similar to the protocol described in our previous work^4^.

### MRI data collection and preprocessing

MRI data were acquired on a Philips Achieva 3 T MRI Scanner in the Affiliated Brain Hospital of Guangzhou Medical University. The functional data were obtained using an echo-planar imaging (EPI) sequence with the following parameters: repetition time (TR) = 9,000 ms, echo time (TE) = 30 ms, flip angle = 90°, field of view (FOV) = 211 mm × 211 mm, data matrix = 64 × 64, voxel size = 3.44 × 3.44 × 4.6 mm^3^. High-resolution brain structural images were obtained using a T1-weighted 3D gradient-echo sequence (TR = 8.2 ms, TE = 3.8 ms, flip angle = 7°, data matrix = 256 × 256, voxel size = 1 × 1 × 1 mm^3^). Each study participant was instructed to keep their eyes closed, to relax but not fall asleep, and to move as little as possible.

The structural data were preprocessed using SPM 12 (https://www.fil.ion.ucl.ac.uk/spm/) and DPABI (version 4.3, http://rfmri.org/dpabi). The GMV was calculated as follows: we first segmented the original T1-weighted images into gray matter (GM), white matter (WM), and cerebrospinal fluid (CSF) images. Then, the segmented GM images for all of the subjects were used to create a customized Diffeomorphic Anatomical Registration using an Exponentiated Lie algebra (DARTEL)^68^ template. Afterward, the GM images were warped to the DARTEL template and spatially normalized to the Montreal Neurological Institute (MNI) space with modulation. Next, the modulated images were smoothed with a Gaussian kernel of 8 mm FWHM^69^. Finally, we extracted the GMV of 90 cerebral regions segmented by the Anatomical Automatic Labeling (AAL) atlas for each subject from the modulated and smoothed images.

The functional data were preprocessed using SPM 12 (https://www.fil.ion.ucl.ac.uk/spm/) and DPABI (version 4.3, http://rfmri.org/dpabi). For each subject, we performed the preprocessing as follows. First, we removed the first 10 time points to eliminate the nonuniform magnetic field and patient inadaptability to the environment. Then, we performed slice timing correction, and the images were realigned to the first volume for head motion correction. Subsequently, we coregistered the functional images to the individual structural images and then normalized them in Montreal Neurological Institute (MNI) standard space by using an affine transformation with the voxels being resampled to 3 × 3 × 3 mm^3^ isotropic voxels. Finally, the resampled data were bandpass (0.01–0.08 Hz) filtered to reduce low-frequency drift and high-frequency physiological noise and spatially smoothed with a Gaussian kernel of 4 mm full width at half maximum (FWHM). After that, we extracted the ReHo and ALFF indexes of each subject from the preprocessed images.

The ReHo index was calculated as follows: first, the ReHo index of each voxel was denoted by Kendall’s coefficient of concordance (KCC) of the time series of this voxel with its 26 nearest neighbors^53^. Then, the raw ReHo index of each voxel was divided by the global mean ReHo index for each subject to reduce the global effects of variability across the participants^70^. Subsequently, the individual ReHo maps were partitioned into 90 cerebral regions by the AAL atlas, and the mean ReHo index of each region was acquired by averaging the ReHo indexes within that region. Finally, we obtained the ReHo index of each brain region segmented by the AAL atlas for each subject.

The ALFF index was calculated as follows: first, each voxel of the time series was converted to the frequency domain by using fast Fourier transformation^33^. Then, the square root of the power spectrum was calculated and averaged across a predefined frequency range. ALFF is the averaged square root, which reflects the absolute intensity of spontaneous brain activity. Finally, the whole brain voxel average ALFF was divided to reduce the global effects of variability across the subjects to achieve standardization^71^. Finally, we obtained the ALFF index of 90 cerebral regions by the AAL atlas for each subject.

### Statistical analyses of bioinformatics and brain function

Sequencing of the V4 region of the 16S rRNA gene was performed on the Illumina MiSeq platform. The sequence data were processed to concatenate reads into tags according to the overlapping relationship by using QIIME2^72^. The raw sequencing results were demultiplexed and quality controlled by applying the DADA2^73^ algorithm to generate feature sequences. The output features were rarefied to 13,500 sequences per sample, which was the lowest value in the dataset. Features containing fewer than 2 sequences or those present in less than 20% of the subjects were filtered out. The microbial community structure was characterized using measures of alpha diversity and beta diversity. The alpha diversity indexes we selected were evenness, Faith’s phylogenetic diversity (Faith_PD), observed species and the Shannon index. Since the sequence number of fecal samples from one SZ subject was lower than the set sampling depth (13,500), this sample was dropped from the alpha diversity analysis. The differences in diversity between groups were calculated using the nonparametric Kruskal–Wallis *H* test in QIIME2. The Bray–Curtis dissimilarity of beta diversity indicates differences in taxa composition between samples based on quantitative species abundance data, which may be presented in a distance matrix. Output matrices were ordinated and visualized using the vegan package from R^74^. A classifier for taxonomy analysis was trained based on sequences and taxonomic results from the Greengenes database (http://greengenes.lbl.gov).

The taxonomic table was normalized to the relative abundances at different taxa levels, and 1321 features and 153 genera were obtained. All differential abundances at the genus level were tested using the Mann–Whitney *U* test. Two-sample *t*-tests were performed on the MRI indexes to compare the differences in functional brain activity between the SZ patients and the NCs. To determine the association between the differential abundance at the genus level and the MRI indexes, we further calculated the residuals of the relative abundances of those taxa and MRI indexes with significant group differences, controlling for age, sex and years of education by the ‘vglm’ function in the VGAM package^75^. Pearson’s correlations were then calculated between the residuals of the relative abundances of the altered genera and the different MRI indexes. The significance of all tests was set at *p* < 0.05 or FDR corrected *p* < 0.05 (two-sided).

### Ethical approval

The study protocol was approved by the ethics committees of the Affiliated Brain Hospital of Guangzhou Medical University. Written informed consent was obtained from each subject before the study.

### Informed consent

Informed consent was obtained from all individual participants included in the study.

## Conclusion

In summary, the current study indicated significant correlations between the gut microbiome and brain structure and function in SZ patients. We found that both Faith_PD and observed species of alpha diversity were significantly correlated with GMV of the bilateral insula and right postcentral gyrus. Additionally, Faith_PD showed a positive correlation with GMV of the left inferior operculum frontal cortex. Both evenness and Shannon indexes of alpha diversity in SZ were positively associated with ReHo indexes of the bilateral calcarine cortex, bilateral lingual gyrus, left superior occipital cortex and right superior parietal cortex. Furthermore, evenness showed positive correlations with ReHo indexes of the right cuneus lobe, bilateral fusiform gyrus, left postcentral gyrus and left superior parietal cortex. Additionally, we found that the ReHo indexes in the right STC, left cuneus and right MTC were negatively correlated with the abundance of the genus *Roseburia*. These findings demonstrated that the properties of the gut microbiome might be associated with alterations in brain structure and function in SZ patients.

## Supplementary Information

Supplementary Information

## Data Availability

The datasets generated and analyzed in the current study are available from the corresponding author upon reasonable request.
