# Author Correction: The gut microbiome is associated with brain structure and function in schizophrenia

**DOI:** 10.1038/s41598-021-96985-2

**Published:** 2021-08-30

**Authors:** Shijia Li, Jie Song, Pengfei Ke, Lingyin Kong, Bingye Lei, Jing Zhou, Yuanyuan Huang, Hehua Li, Guixiang Li, Jun Chen, Xiaobo Li, Zhiming Xiang, Yuping Ning, Fengchun Wu, Kai Wu

**Affiliations:** 1 Department of Biomedical Engineering, School of Material Science and Engineering, South China University of Technology, Guangzhou, 510006 China; 2 The Affiliated Brain Hospital of Guangzhou Medical University, Guangzhou Huiai Hospital, Guangzhou, 510370 China; 3 Guangdong Engineering Technology Research Center for Translational Medicine of Mental Disorders, Guangzhou, 510370 China; 4 Guangdong Engineering Technology Research Center for Diagnosis and Rehabilitation of Dementia, Guangzhou, 510500 China; 5 National Engineering Research Center for Tissue Restoration and Reconstruction, South China University of Technology, Guangzhou, 510006 China; 6 Key Laboratory of Biomedical Engineering of Guangdong Province, South China University of Technology, Guangzhou, 510006 China; 7 National Engineering Research Center for Healthcare Devices, Guangzhou, 510500 China; 8 Department of Biomedical Engineering, New Jersey Institute of Technology, Newark, NJ USA; 9 Department of Radiology, Panyu Central Hospital of Guangzhou, Guangzhou, 511400 China; 10 Department of Nuclear Medicine and Radiology, Institute of Development, Aging and Cancer, Tohoku University, Sendai, 980‑8575 Japan

Correction to: *Scientific Reports* 10.1038/s41598-021-89166-8, published online 07 May 2021

The original version of this Article contained an error in the caption of vertical coordinate in Figure 4(b). As a result,

“Alpha diversity index (v)”

now reads:

“Relative abundance of Roseburia (v)”.

The original Figure 4 and accompanying legend appear below.

**Figure 4:**
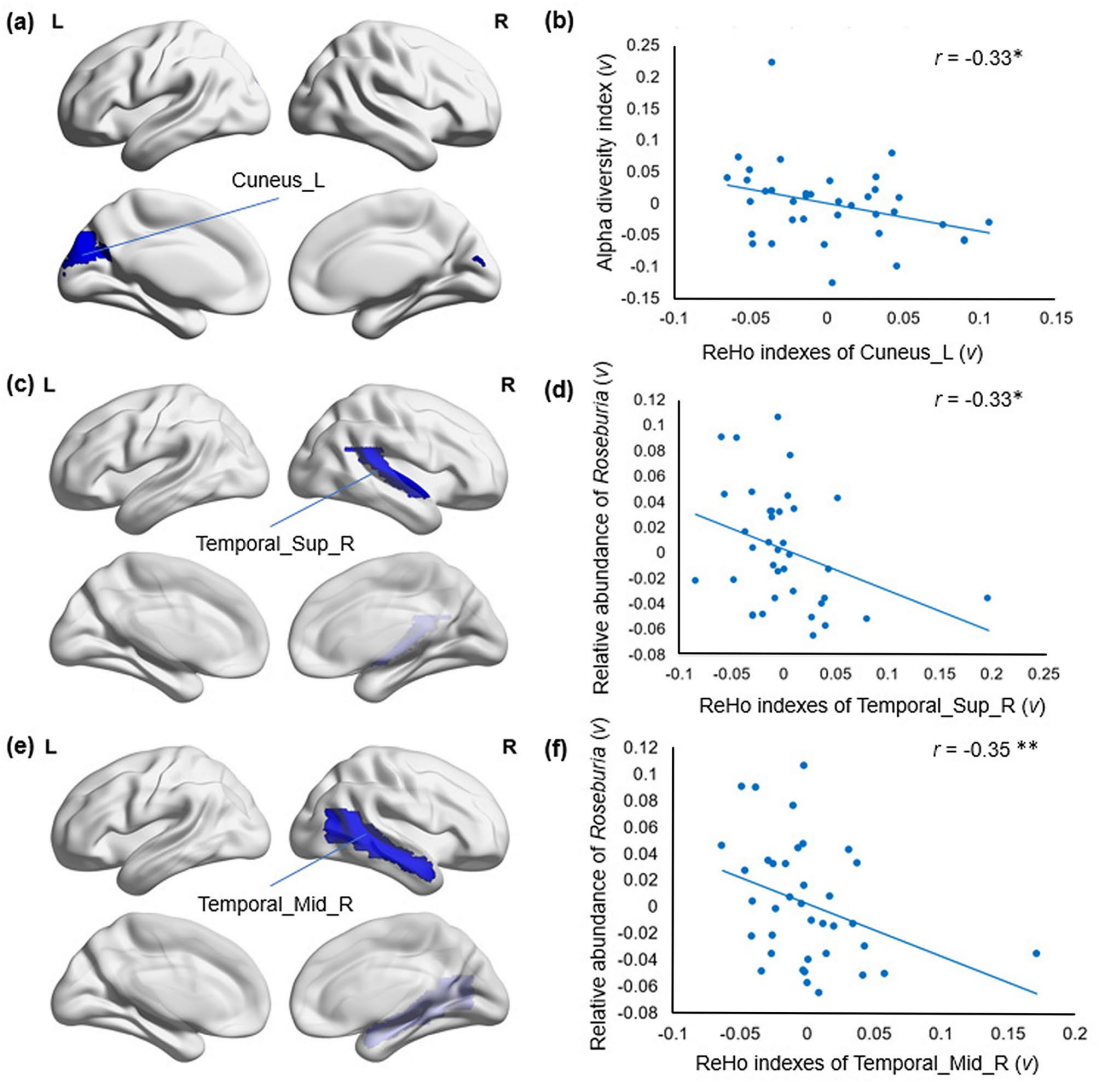
Relative abundance of *Roseburia* in SZ patients showed strong negative correlations with ReHo indexes. (**a**) Cuneus_L showed significantly decreased ReHo indexes in SZ compared with the NCs (*p* < 0.05). (**b**) The residuals of the ReHo indexes in Cuneus_L were significantly negatively correlated with the residuals of the relative abundance of Roseburia in SZ. (**c**) Temporal_Sup_R showed significantly decreased ReHo indexes in SZ compared with the NCs (*p* < 0.05). (**d**) The residuals of the ReHo indexes in Temporal_Sup_R were significantly negatively correlated with the residuals of the relative abundance of *Roseburia* in SZ. (**e**) Temporal_Mid_R showed significantly decreased ReHo indexes in the SZ compared with the NCs (*p* < 0.05). (**f**) The residuals of ReHo indexes in Temporal_Mid_R were significantly negatively correlated with the residuals of the relative abundance of *Roseburia* in SZ. Sup: superior; Inf: inferior; L: left hemisphere; R: right hemisphere. **p < 0.05, FDR corrected, *p = 0.05, FDR corrected. Figure was generated by a brain network visualization tool of “BrainNet viewer” (Version 1.7, https://www.nitrc.org/projects/bnv/), based on MATLAB (Version 2017a).

The original Article has been corrected.

