# Supplementary material for: The gut microbiome is associated with brain structure and function in schizophrenia
Source: Sci Rep. 2021 May 7;11:9743. doi: 10.1038/s41598-021-89166-8 (PMC8105323; doi:10.1038/s41598-021-89166-8)
Supplement: Supplementary file 1 — Supplementary Information [file 41598_2021_89166_MOESM1_ESM.docx]

**Association between the gut microbiome and functional brain activity in schizophrenia-Supplementary Materials**

Shijia Li ^a, c, e, †^, Jie Song ^a, c, e, †^, Pengfei Ke ^a, c, e^, Lingyin Kong ^a, c, e^, Bingye Lei ^a, c, e^, Jing Zhou ^a, c, e^, Yuanyuan Huang ^b, c^, Hehua Li ^b, c^, Guixiang Li ^d, g^, Jun Chen ^d, g^, Xiaobo Li ^c, h^, Zhiming Xiang ^d, I^, Yuping Ning ^b, c^, Fengchun Wu ^b, c,^ * and Kai Wu ^a,b,c,d,e,f,g,j,^*

^a^ Department of Biomedical Engineering, School of Material Science and Engineering, South China University of Technology, Guangzhou 510006, China

^b^ The Affiliated Brain Hospital of Guangzhou Medical University, Guangzhou Huiai Hospital, Guangzhou 510370, China

^c^ Guangdong Engineering Technology Research Center for Translational Medicine of Mental Disorders, Guangzhou 510370, China

^d^ Guangdong Engineering Technology Research Center for Diagnosis and Rehabilitation of Dementia, Guangzhou 510500, China

^e^ National Engineering Research Center for Tissue Restoration and Reconstruction, South China University of Technology, Guangzhou 510006, China

^f^ Key Laboratory of Biomedical Engineering of Guangdong Province, South China University of Technology, Guangzhou 510006, China

^g^ National Engineering Research Center for Healthcare Devices, Guangzhou 510500, China

^h^ Department of Biomedical Engineering, New Jersey Institute of Technology, Newark, NJ, U.S.

^i^ Department of Radiology, Panyu Central Hospital of Guangzhou, Guangzhou 511400, China

^j^ Department of Nuclear Medicine and Radiology, Institute of Development, Aging and Cancer, Tohoku University, Sendai 980-8575, Japan

^†^ Shi-jia Li and Jie Song contributed equally to this work.

* Corresponding authors: [kaiwu@scut.edu.cn](mailto:kaiwu@scut.edu.cn) (K. Wu.); [13580380071@163.com](mailto:13580380071@163.com) (F.-c. Wu).

Supplementary Table S1. Alpha diversity in the NC and SZ groups

| Alpha diversity | NC group | SZ group | *p* |
| --- | --- | --- | --- |
| Shannon | 4.34±0.70 | 4.17±0.75 | 0.29 |
| Evenness | 0.65±0.08 | 0.64±0.09 | 0.68 |
| Faith’s phylogenetic diversity | 8.06±2.04 | 7.72±1.96 | 0.45 |
| Observed features | 103.47±34.11 | 95.78±32.54 | 0.41 |

**Notes:** NC, normal control; SZ, schizophrenia.

Supplementary Table S2. Difference of MRI indexes between the NC and SZ groups

| MRI | Index | Regions | NC group | SZ group | *p* |
| --- | --- | --- | --- | --- | --- |
| GMV | 11 | Frontal_Inf_Oper_L | 0.42±0.04 | 0.39±0.05 | 0.04 |
|  | 25 | Frontal_Mid_Orb_L | 0.45±0.05 | 0.41±0.07 | 0.02 |
|  | 29 | Insula_L | 0.54±0.04 | 0.50±0.06 | 0.03 |
|  | 30 | Insula_R | 0.56±0.04 | 0.53±0.06 | 0.03 |
|  | 41 | Amygdala_L | 0.64±0.05 | 0.61±0.07 | 0.03 |
|  | 44 | Calcarine_R | 0.43±0.05 | 0.40±0.06 | 0.04 |
|  | 49 | Occipital_Sup_L | 0.33±0.03 | 0.31±0.04 | 0.04 |
|  | 51 | Occipital_Mid_L | 0.42±0.03 | 0.39±0.05 | 0.03 |
|  | 58 | Postcentral_R | 0.32±0.03 | 0.30±0.04 | 0.04 |
|  | 73 | Putamen_L | 0.45±0.06 | 0.42±0.07 | 0.05 |
|  | 74 | Putamen_R | 0.43±0.05 | 0.40±0.06 | 0.01 |
|  | 77 | Thalamus_L | 0.41±0.03 | 0.39±0.04 | 0.03 |
|  | 78 | Thalamus_R | 0.44±0.03 | 0.41±0.05 | 0.04 |
|  | 83 | Temporal_Pole_Sup_L | 0.33±0.03 | 0.31±0.04 | 0.01 |
|  | 84 | Temporal_Pole_Sup_R | 0.34±0.03 | 0.31±0.04 | 0.01 |
|  | 90 | Temporal_Inf_R | 0.49±0.05 | 0.46±0.07 | 0.03 |
| ReHo | 1 | Precentral_L | 0.43±0.04 | 0.41±0.06 | 0.05 |
|  | 13 | Frontal_Inf_Tri_L | 0.47±0.03 | 0.45±0.05 | 0.03 |
|  | 14 | Frontal_Inf_Tri_R | 0.46±0.03 | 0.44±0.06 | 0.05 |
|  | 2 | Precentral_R | 0.44±0.04 | 0.42±0.05 | 0.05 |
|  | 25 | Frontal_Mid_Orb_L | 0.52±0.05 | 0.49±0.06 | 0.02 |
|  | 26 | Frontal_Mid_Orb_R | 0.51±0.04 | 0.48±0.06 | 0.004 |
|  | 27 | Rectus_L | 0.48±0.08 | 0.44±0.05 | 0.01 |
|  | 28 | Rectus_R | 0.48±0.07 | 0.44±0.05 | 0.01 |
|  | 43 | Calcarine_L | 0.55±0.08 | 0.50±0.08 | 0.003 |
|  | 44 | Calcarine_R | 0.54±0.07 | 0.49±0.08 | 0.01 |
|  | 45 | Cuneus_L | 0.55±0.06 | 0.51±0.07 | 0.01 |
|  | 46 | Cuneus_R | 0.54±0.05 | 0.51±0.07 | 0.02 |
|  | 47 | Lingual_L | 0.50±0.06 | 0.45±0.07 | 0.004 |
|  | 48 | Lingual_R | 0.50±0.06 | 0.45±0.08 | 0.002 |
|  | 49 | Occipital_Sup_L | 0.47±0.04 | 0.44±0.07 | 0.01 |
|  | 5 | Frontal_Sup_Orb_L | 0.45±0.06 | 0.43±0.06 | 0.04 |
|  | 50 | Occipital_Sup_R | 0.50±0.04 | 0.46±0.07 | 0.01 |
|  | 51 | Occipital_Mid_L | 0.51±0.04 | 0.48±0.06 | 0.01 |
|  | 52 | Occipital_Mid_R | 0.53±0.04 | 0.50±0.07 | 0.01 |
|  | 53 | Occipital_Inf_L | 0.49±0.05 | 0.45±0.07 | 0.01 |
|  | 54 | Occipital_Inf_R | 0.52±0.06 | 0.47±0.08 | 0.002 |
|  | 57 | Postcentral_L | 0.43±0.05 | 0.40±0.06 | 0.01 |
|  | 58 | Postcentral_R | 0.46±0.05 | 0.42±0.07 | 0.002 |
|  | 59 | Parietal_Sup_L | 0.46±0.05 | 0.43±0.06 | 0.01 |
|  | 6 | Frontal_Sup_Orb_R | 0.45±0.05 | 0.42±0.05 | 0.02 |
|  | 60 | Parietal_Sup_R | 0.47±0.04 | 0.44±0.06 | 0.003 |
|  | 61 | Parietal_Inf_L | 0.46±0.04 | 0.43±0.06 | 0.04 |
|  | 62 | Parietal_Inf_R | 0.51±0.04 | 0.48±0.06 | 0.01 |
|  | 67 | Precuneus_L | 0.54±0.04 | 0.51±0.06 | 0.04 |
|  | 71 | Caudate_L | 0.37±0.02 | 0.39±0.06 | 0.05 |
|  | 8 | Frontal_Mid_R | 0.48±0.03 | 0.46±0.06 | 0.03 |
|  | 81 | Temporal_Sup_L | 0.45±0.04 | 0.42±0.06 | 0.03 |
|  | 82 | Temporal_Sup_R | 0.48±0.04 | 0.45±0.05 | 0.03 |
|  | 86 | Temporal_Mid_R | 0.49±0.03 | 0.46±0.05 | 0.02 |
| ALFF | 72 | Caudate_R | 0.76±0.15 | 0.93±0.5 | 0.04 |

**Notes:** GMV, gray matter volume; ReHo, regional homogeneity, ALFF, amplitude of low-frequency fluctuation; NC, normal control; SZ, schizophrenia; Mid, middle; Inf, inferior; Oper, operculum; Tri, triangularis; Orb, orbitalis; Sup, superior; L (R), left (right) hemisphere.

Supplementary Table S3. Relationship between the diversity of microbiome and MRI indexes in SZ patients

| Alpha diversity | Regions | *r* | *p* | *p­** |
| --- | --- | --- | --- | --- |
| Observed species | Postcentral_R | 0.41 | 0.011 | 0.024 |
|  | Insula_R | 0.41 | 0.012 | 0.026 |
|  | Insula_L | 0.40 | 0.016 | 0.032 |
| Faith_PD | Insula_L | 0.45 | 0.005 | 0.013 |
|  | Insula_R | 0.45 | 0.005 | 0.013 |
|  | Postcentral_R | 0.42 | 0.010 | 0.023 |
|  | Frontal_Inf_Oper_L | 0.40 | 0.015 | 0.030 |
| Evenness | Calcarine_R | 0.59 | 0.000 | 0.000 |
|  | Calcarine_L | 0.49 | 0.002 | 0.003 |
|  | Lingual_R | 0.48 | 0.003 | 0.004 |
|  | Occipital_Sup_L | 0.48 | 0.003 | 0.004 |
|  | Lingual_L | 0.47 | 0.003 | 0.004 |
|  | Cuneus_R | 0.40 | 0.013 | 0.017 |
|  | Occipital_Inf_L | 0.38 | 0.020 | 0.025 |
|  | Parietal_Sup_L | 0.38 | 0.021 | 0.027 |
|  | Occipital_Inf_R | 0.37 | 0.025 | 0.032 |
|  | Postcentral_L | 0.36 | 0.028 | 0.036 |
|  | Parietal_Sup_R | 0.36 | 0.028 | 0.036 |
| Shannon | Calcarine_R | 0.56 | 0.000 | 0.000 |
|  | Calcarine_L | 0.47 | 0.003 | 0.004 |
|  | Lingual_L | 0.43 | 0.008 | 0.011 |
|  | Occipital_Sup_L | 0.42 | 0.010 | 0.013 |
|  | Lingual_R | 0.42 | 0.010 | 0.013 |
|  | Parietal_Sup_R | 0.36 | 0.031 | 0.039 |

**Notes:** Sup, superior; Inf, inferior, L (R), left (right) hemisphere; *p**, *p* value with FDR correction.

Supplementary Table S4. Relationship between the MRI indexes and PANSS scores in SZ patients

| PANSS | MRI | Index | Regions | *r* | *p* | *p­** |
| --- | --- | --- | --- | --- | --- | --- |
| Positive | GMV | 11 | Frontal_Inf_Oper_L | 0.45 | 0.005 | 0.013 |
|  |  | 58 | Postcentral_R | 0.41 | 0.011 | 0.025 |
| Negative | DC | 21 | Olfactory_L | 0.37 | 0.023 | 0.034 |
| General | DC | 21 | Olfactory_L | 0.40 | 0.014 | 0.021 |
|  |  | 22 | Olfactory_R | 0.36 | 0.026 | 0.038 |
| Total | DC | 21 | Olfactory_L | 0.40 | 0.013 | 0.020 |
|  |  | 22 | Olfactory_R | 0.38 | 0.019 | 0.028 |
|  |  | 73 | Putamen_L | 0.35 | 0.031 | 0.042 |
|  |  | 83 | Temporal_Pole_Sup_L | 0.36 | 0.029 | 0.042 |

**Notes:** Inf, inferior; Oper, operculum; Sup, superior; L (R), left (right) hemisphere; *p**, p value with FDR correction.
